# Factors Influencing Mood Disorders and Health Related Quality of Life in Adults With Glioma: A Longitudinal Study

**DOI:** 10.3389/fonc.2021.662039

**Published:** 2021-05-20

**Authors:** Antonella Leonetti, Guglielmo Puglisi, Marco Rossi, Luca Viganò, Marco Conti Nibali, Lorenzo Gay, Tommaso Sciortino, Henrietta Howells, Luca Fornia, Marco Riva, Gabriella Cerri, Lorenzo Bello

**Affiliations:** ^1^ Laboratory of Motor Control, Department of Medical Biotechnologies and Translational Medicine, Università degli Studi di Milano, Milano, Italy; ^2^ Neurosurgical Oncology Unit, Department of Oncology and Hemato-Oncology, Università degli Studi di Milano, Milano, Italy; ^3^ Neurosurgical Oncology Unit, Department of Medical Biotechnologies and Translational Medicine, Università degli Studi di Milano, Milano, Italy; ^4^ BIOMETRA Department, Humanitas Research Hospital IRCSS, Milano, Italy

**Keywords:** mood disorders, health related quality of life, brain tumors, recovery, adjuvant treatments

## Abstract

**Objective:**

At present, it is not clear whether Mood Disorders (MD) and poor Health Related Quality of Life (HRQoL) in the glioma population correlate with features of the tumor, or rather with secondary symptoms associated with treatment. The aim of this study was to assess the prevalence of MD and decline in HRQoL in glioma patients, and to determine the main factors associated with these two variables.

**Methods:**

80 patients affected by lower-grade gliomas (LGGs) and 65 affected by high-grade gliomas (HGGs) were evaluated, from admission up to 12 months after surgery, for MD, HRQoL, clinical characteristics, and cognitive functions. Independent factors associated with MD and low HRQoL were identified by using bivariate analysis.

**Results:**

Data showed that prevalence of low HRQoL was comparable in both groups during all the time points assessed (pre, 1, 3, 6 and 12 months after surgery). In contrast at 6 months following surgery, HGGs showed a higher prevalence of MD compared to LGGs;. Bivariate analysis revealed that factors associated with MD and HRQoL in LGGs and HGGs were different over the course of the disease. In LGGs, from the pre-operative period to one year post surgery, MD and low HRQOL were associated with the occurrence of cognitive deficits and, from the third month after surgery onward, they were also associated with the effect exerted by adjuvant treatments. In HGGs, MD were associated with cognitive deficits at 3 and 6 months after surgery, along with older age (65-75 years); HRQoL, in its Physical component in particular, was associated with older age only from 6 months after surgery.

**Conclusion:**

Factors associated with MD and low HRQoL were different in LGGs and HGGs over the course of the disease. In LGGs the effect of adjuvant treatments was prominent in determining the prevalence of both MD and poor HRQoL from the third month after surgery onward. In HGGs, MD and HRQoL were associated with age, at 3 and 6 months after surgery. In both, the occurrence of cognitive deficits was significantly associated with MD.

## Introduction

Gliomas are primary brain tumors that have a variable prognosis. Survival ranges widely, for patients with glioblastoma (15 months-2 years), other high-Grade Gliomas (HGGs) such as astrocytoma or oligodendroglioma anaplastic (3-10 years) or lower-grade gliomas (LGGs; 7-15 years) (1). Such variance depends on several factors, such as the biological behavior of the tumors, age of the patient, and treatment(s) adopted (2). Independent of prognosis, the diagnosis of brain tumor itself may create stress and significantly affects a person’s life due to the awareness of having a potentially fatal condition and to the complex treatments to be potentially/possibly adopted, along with its resulting side effects (3). Up to approximately 80% of brain tumor patients experience a variety of neurological or cognitive symptoms during the course of the disease (4–6), especially after treatments (7–9). Along with significant neurocognitive impairment, patients affected by brain tumors are also more likely to develop reactive mood disorders (MD) such as depression and/or anxiety, compared with healthy subjects or patients affected by other cancers not directly involving the central nervous system (CNS) (3, 10). MD is reported to range between 2.8% to 95% for depression, and between 13% to 60% for anxiety (11–13). Such wide variance may be associated with multiple factors, either clinical (treatment, histology, location) or functional (neurological and cognitive dysfunctions). Taken together, the alterations in physical, cognitive and psychological status, along with the side effects of the treatments can dramatically impact on the health related quality of life (HRQoL): i.e. “the subjective perception, influenced by the current health status (physical and mental) of the ability to cope with those activities important for the individual” (14). In fact, 45% of patients with a lower-grade glioma report a low global (poor) quality of life, with fewer than half of patients being able to carry out daily life activities without restriction (15).

Rather than depending on the grade of tumor, the effect on quality of life seems to be more related to the course of the tumor, whether it is stable or progressive, this supported by data suggesting that both LGG and HGG patients show a similar HRQoL when the stability of the clinical course is assured (16). However, in most cases there is a significant difference in prognosis and treatment, and patients with HGGs or LGGs may experience different treatment pathways (i.e. radiotherapy and closer follow-up for HGGs) that are unavoidably characterized by different distress profiles. Currently, tumor location (site and lobe), tumor aggressiveness, local disease control and adjuvant treatments are all considered to be factors associated with the development of mood disorders (17, 18), and are also crucial determinants of HRQoL (19).

Based on the evidence suggesting that both MD and HRQoL are associated with a short survival rate and poor medical compliance (7, 20, 21), there has been a progressive scientific effort in investigating factors affecting mood and quality of life in glioma patients.

However, despite the increasing number of studies focusing on these aspects, at present, available literature reports contradictory findings about the possible association of clinical and biological variables with the onset of MD and HRQoL, and particularly for MD, no definitive conclusions can yet be drawn (22, 23). In addition, separate data on low or high-grade gliomas are currently very limited or lacking (24).

Based on this premise, quantification of the prevalence of MD and poor HRQoL and identification of the main factors associated with these conditions in LGG and HGG patients is relevant, in order to provide an unbiased evaluation of patient’s cancer care outcomes (25). In this longitudinal study, we measured the prevalence of MD and of decline in HRQoL in a large sample of LGG and HGG patients, and we evaluated the main associated factors, focusing on demographic, clinical, anatomical, histological and functional variables, from diagnosis till 12 months after surgery. The aim was to quantify the prevalence of MD and low HRQoL and to identify, during the first year of the disease, the factors associated with the risk of developing MD or a decline in HRQoL. The identification of such predictors will help in planning specific interventions tailored to patients’ specific conditions, aimed at improving prognosis and care.

## Materials and Methods

### Participants and Data Collection

We prospectively enrolled 238 subjects selected from individuals consecutively admitted between January 2017 and October 2018 for a glioma resection at our Neurosurgical Oncology Unit. Inclusion criteria were: I) age ≥ 18 years; II) absence of severe comprehension deficits affecting the abilities to complete the questionnaires (comprehension abilities were tested before the administered MD and HRQoL questionnaires using standardized test (Token test) at each timepoint; III) absence of previous psychiatric symptoms or disease; IV) absence of current medications for psychiatric conditions; V) histo-molecular diagnosis of LGGs and HGGs and VI) newly diagnosed glioma with no history of treatments (surgery, chemo or radio – therapies). The LGG group included patients with a grade II and III IDH-mutated tumors; the HGG group, patients with a grade III-IV IDH-wildtype or IV IDH-mutated tumors. Tumor aggressiveness was classified upon histo-molecular profile.

The exclusion criterion was the absence of tumor progression during the assessment period, as evaluated by follow-up MRI and clinical evaluations or Tumor Board Discussion. If, at the time of the MD and HRQoL assessment (t0, t1, t2, t3 and t4), a progression of the disease was diagnosed, the patient was excluded even if he/she had performed any previous assessments. Based on the inclusion and exclusion criteria, 93 patients were excluded at the end of the study. The final sample was composed by 145 patients (see **Table 1** for the clinical and demographics characteristics).

**Table 1:** Frequency of the clinical and demographic characteristics of Lower (LGG) and High Grade Glioma (HGG) groups.

	LGG group (N 80)		HGG group (N. 65)		Significance (X^2^)
CLINICAL VARIABLES	%	N	%	N	
**Histological Profile**					
Astrocytoma	30,0	24	/	/	
Oligodendroglioma	28,75	23	/	/	
Gangoglioma	17,5	14	/	/	
Anaplastic Astrocitoma	/	/	12,3	8	
Anaplastic Oligodendroglioma	/	/	18,5	12	
Glioblastoma	/	/	69,2	45	
Other	23,75	19	/	/	
**IDH**					0.001
Mutate	73,8	59	33,8	22	
Wildtype	26,3	21	66,2	43	
**HEMISPHERIC LATERALITY**					0.335
*Right*	53,8	43	33,8	22	
*Left*	46,3	37	66,2	43	
**LOBE AFFECTED**					0.107
*Frontal*	51,2	41	27,7	18	
*Insular*	18,2	15	10,8	7	
*Temporal*	12,5	10	33,8	22	
*Parietal*	16,3	13	23,1	15	
*Other*	1,3	1	4,6	3	
**ADJUVANT THERAPY**					0
*Chemotherapy*	57,5	46	98,5	64	
*Radiotherapy*	52,5	42	98,5	64	
**Gender**					0.33
*Male*	57,5	46	64,6	42	
*Female*	42,5	34	35,4	23	
**Age**					0
*Mean (SD)*	39,70 (11,3)		51,2 (13,3)		
**Education**					0.975
*Mean (SD)*	13,9 (3,01)		13,7 (3,25)		

Clinical and demographics characteristics of Low Grade Glioma (LGG) and High Grade Glioma (HGG) groups. For each group and for each variables the percentage (%) total number (N) of subjects and X^2^ was reported.

For each patient, the clinical records were reviewed and the relevant data relative to tumor (type, grade, IDH-mutation, location), medications (anti-epileptic drugs -AEDs- and steroids) given before and during treatments, and adjuvant therapies (chemotherapy and radiotherapy) were recorded. For each patient we also reported the socio-demographics characteristics (age and level of education), personal or family history of psychiatric disorder and current or previous treatments with psychotropic medication. All patients gave written informed consent to the surgical and clinical procedure (IRB1299), which followed the principles outlined in the declaration of Helsinki.

### Demographic and Clinical Features

One hundred and forty-five patients were enrolled. Sixty-four were HGGs and 81 LGGs. 60.7% were males; the frontal lobe (40.7%) and the left hemisphere (55.2%) were more frequently affected. 88.3% underwent a total resection and 11.7% a supra-total resection; 65.3% of patients (96.9% of HGGs and 40% of LGGs) received radiotherapy, starting 1 month after surgery, followed by adjuvant chemotherapy (Temozolamide-based standard regimen; average number of cycles: 9 (range 0-9) in HGGs and 6 (range 0-9) in LGGs. The choice of submitting a patient to adjuvant treatment was performed by Tumor Board upon histo-molecular phenotype. The majority of patients in both groups (90% of HGGs and 88% of LGGs) had seizures the year before surgery, while only 10% of HGGs and 7% of LGGs experienced (focal) seizures during the 12-months follow-up assessment. All patients were on antiepileptic drugs -AEDs - (Lacosamide and/or Levetiracetam) during the course of the disease; all patients used AEDs from the pre-operative period to 12 month after surgery, while only 50% of LGGs patients stopped treatment at 1 year. Steroids were used in all patients in the pre-operative period (desametasone, average 2 mg/day before surgery) and in the post-operative for an average of 13 days (inclusive of tapering) (average 4 mg/day); steroids were again given during the course of radiotherapy and tapered off after 10 days (average 8 mg/day). Clinical, and demographic characteristics of the participants are summarized in **Table 1**.

### Study Design

All patients were submitted to a preoperative (1 week before surgery - t0) and four post-operative (1 month (t1), 3 months (t2), 6 months (t3) and 12 months (t4) after surgery) extensive assessments.

At each time point, patients’ functional outcomes were assessed by neurological evaluation and standardized neuropsychological tests for five domains: language, praxis abilities, attentive, executive and memory functions (**Table 2**). Based on the assessment, we recorded: 1) neurological deficits (motor and visual impairment) and 2) cognitive deficits. In each patient the raw score of each test was corrected for patient age and educational level to obtain a normalized score subsequently compared with the distribution of scores of an Italian control population: scores falling into the worst 5th percentile of the scores observed in the controls were defined as pathological [see (26) for an extensive description of the procedure]. A specific cognitive domain was considered to be impaired when at least one of the corresponding test scores was defined as pathological [for similar approach see (27, 28)].

**Table 2:** Neuropsychological assessment.

DOMAINS	Test
***LANGUAGE***	-Token Test
	-Picture Naming test
	-Verbal Fluency (Phonemic and semantic)
***ATTENTION AND EXECUTIVE FUNCTIONS***	-Attentive Matrice
	-Trail Making Test
	-Stroop Test
***MEMORY***	-Digit span backward
	-Rey’s Auditory Verbal Learning Test
	-Recall Rey figure
***PRAXIS***	-Ideomotor apraxia test
	-Oro-facial apraxia test

For each cognitive domain the test used is reported.

### Outcome Measures

At each time point, patients were also assessed with two self-report questionnaires evaluating whether the patient was experiencing MD (anxiety and depression symptoms) or poor HRQoL.

1. Among the available screening tools for the evaluation of MD in cancer population the “Hospital Anxiety and Depression Scale” [HADS; (29)] was used. HADS contain fourteen-items assessing Anxiety and Depression symptoms. Patients were asked to check the sentence that best represents their feelings at the time of evaluation. Each item scored from 0 to 3 (overall score 42 points). A cut-off total score of ≥ 10 identified moderate symptoms of MD in accordance with the Italian version (30). The prevalence of mood disorders based on the HADS total cut-off scores was recorded and analyzed statistically.
2. HRQoL was evaluated by using the “Short Form 36 items Health survey (SF36)” (31) of the Medical Outcomes Study (MOS), a largely validated instrument for assessing HRQoL. SF36 was found reliable for assessing HRQoL in the general population and in oncological patients, including those with brain cancer (32). It consists of 36 questions measuring two different dimensions: the Physical Component Summary (PCS) assessing the role limitations due to physical problems, pain and general health, and the Mental Component Summary (MCS) assessing the role limitations caused by emotional problems, vitality, social functioning, and mental health (see 31 for scoring techniques). The PCS and MCS scores are easier to interpret statistically than the eight subscale scores, due to smaller confidence intervals, and lower floor and ceiling effects. The total scores for both components ranged from 0 to 100 and higher scores indicate better HRQoL. By following the procedure previously used to assess outcome in the oncological population^3^, the scores of the two components were compared with the average score recorded in the general control population (see 31 for an extensive description of the statistical procedure). Patients with a score of one standard deviation (SD: 10) below the average score of the Italian control group (PCS: 50 MCS: 49.6) were classified as patients with low HRQoL (31).

### Statistical Analyses

**Figure 1:**
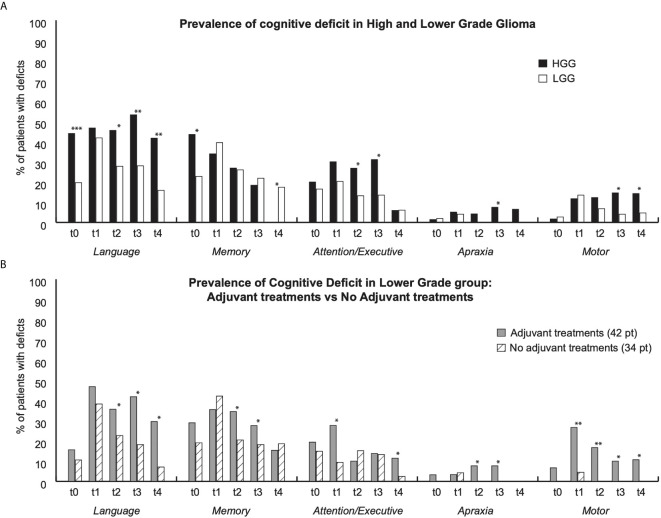
Figure shows prevalence of functional deficits for each group and for each time points (t0: pre surgery; t1:1month after surgery; t2: 3 months after surgery; t3: 6 months after surgery; t4: 12 months after surgery). **(A)** Prevalence of deficit in High Grade glioma (HGG) (Black bars) and in lower grade (White Bars); **(B)** Prevalence of deficits in Lower patients who underwent adjuvant treatments (grey bars) vs patients who did not undergo adjuvant treatment (white dashed bars). * indicate p < 0,05; ** p < 0,01; *** p < 0.001.

**Figure 2:**
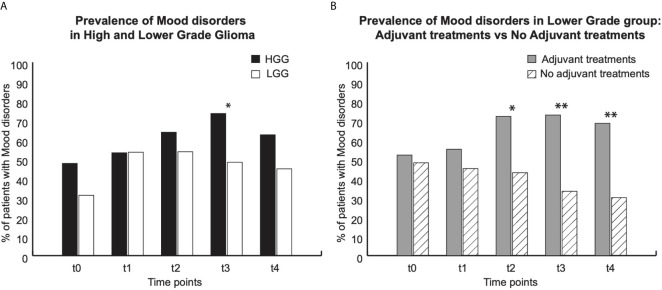
Prevalence of Mood disorders (HADS score). For each group, graphs show data at each time point: t0 (pre surgery); t1 (1 month after surgery); t2, t3 and t4 (respectively 3, 6 and 12 months after surgery). **(A)** Prevalence of MD in High grade Glioma (HGG) (Black bars, and in lower grade glioma (white bars). **(B)** Prevalence of MD in lower grade group: gray bars patients underwent to adjuvant treatments, white dashed bars patients who did not undergo adjuvant treatments. * indicate p < 0,05; ** p < 0,01.

**Figure 3:**
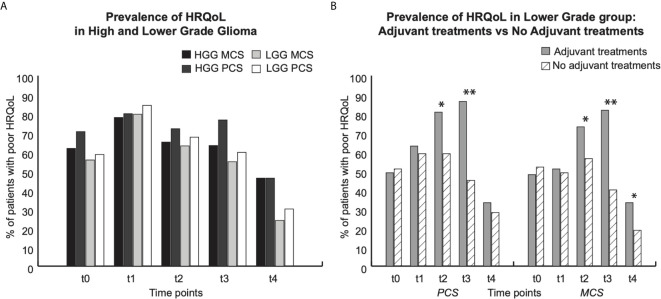
Prevalence of low HRQoL (SF36 score). For each group, graphs show data at each time point: t0 (pre surgery); t1 (1 month after surgery); t2, t3 and t4 (respectively 3, 6 and 12 months after surgery). * indicate p < 0,05; ** p < 0,01. **(A)** Prevalence of low HRQoL in High (black and dark grey bars) and Lower (light grey and white bras) group. Dark Black and light grey bars show prevalence of Mental component score (MCS); light black and white bars show prevalence of Physical component score (PCS). **(B)** Prevalence of low HRQoL in lower grade group: Grey bars patients who underwent adjuvant treatments, dashed white bars: patients who did not undergo adjuvant treatments.

Statistical analyses were performed using IBM SPSS Statistics Software 20. Descriptive statistics were used for the clinical and demographic features of the sample. Differences in prevalence of cognitive deficits, MD, and low HRQoL between LGG and HGG groups, and between lower grade patients treated and untreated with adjuvant therapy, were assessed for each time point (t0, t1, t2, t3 and t4) by 2x2 Chi Square tests (see **Figures 1**–**3**). In both LGG and HGG groups, the association of different functional and clinical variables with MD and HRQoL was investigated by bivariate analyses: for each time point (t0, t1, t2, t3 and t4), 2x2 Chi Square tests was performed to test association between mood disorder (MD), the Physical component of HRQoL (PCS) or Mental Components of HRQoL (MCS) and demographics, clinical, histo-molecular factors (age, sex, education level, affected lobe and hemisphere, adjuvant therapies adopted and IDH mutation) and functional factors (cognitive and neurological deficits) of each group (LGGs *vs* HGGs). Significant associations are shown in **Table 3** and **4**. In accordance with Chi-squared Test assumptions, the associations showing expected cell frequencies smaller than five were not considered (33). Given the exploratory nature of the study, in order to avoid dramatically increase the chance of Type II errors we did not apply corrections for multiple comparisons (34, 35).

**Table 3:** Factors associated with mood disorders in lower (LGGs) and High Grade Glioma (HGG).

	LOWER GRADE GROUP					HIGH GRADE GROUP			
	Factor associated	Prevalence of MD %	X^2^(df=1)	P value		Factor associated	Prevalence of MD %	X^2^(df=1)	P value
***t0***	**Language**		4,305	0,038	***t0***	**–**	**–**		**–**
	Deficit	60				**–**	**–**		**–**
	Normal	24				**–**	**–**		**–**
***t1***	**Motor**		4,002	0,045	***t1***	**–**	**–**		**–**
	Deficit	88				**–**	**–**		**–**
	Normal	50				**–**	**–**		**–**
***t2***	**Language**		3,625	0,046	***t2***	**Language**		4,359	0,037
	Deficit	80				Deficit	84		
	Normal	44				Normal	54		
	**Adjuvant Treatments**		5,354	0,021		**Affected Hemisphere**		7,430	0,011
	Yes	70				Left	70		
	No	40				Right	31		
***t3***	**Language**		5,617	0,018	***t3***	**Language**		3,758	0,048
	Deficit	72				Deficit	90		
	Normal	39				Normal	48		
	**Adjuvant Treatments**		8.487	0,004		**Attentive/Executive**		4,306	0,046
	Yes	74				Deficit	100		
	No	32				Normal	67		
						**Age**		8,036 *(df=2)*	0,038
						Young	20		
						Adult	33		
						Old	67		
***t4***	**Language**		5,939	0,015	***t4***	**–**	**–**		**–**
	Deficit	80				**–**	**–**		**–**
	Normal	37				**–**	**–**		**–**
	**Attentive/Executive**		3,968	0,046		**–**	**–**		**–**
	Deficit	100				**–**	**–**		**–**
	Normal	67				**–**	**–**		**–**
	**Adjuvant Treatments**		6,941	0,008		**–**	**–**		**–**
	Yes	69				**–**	**–**		**–**
	No	30				**–**	**–**		**–**

For each time points (t0: pre surgery; t1:1 month after surgery; t2:3 months after surgery; t3. 6 months after surgery; t4: 1 year after surgery) and for each group (Low grade group (LGG) in the left part; and High grade glioma (HGG) on the right side) factors associated with prevalence of Mood Disorders (MD), was reported. Blank cells: no factors were statistically associated with the prevalence of MD. df, degree of freedom; *X^2^*, *chi square value.*

**Table 4:** Factors Associated with low HRQoL in High and Lower grade glioma group.

HIGH GRADE GROUP								
Mental Component Score					Physical Component Score			
	Factors Associated	Prevalence of low MCS %	X^2^(df=1)	P value	Factors Associated	Prevalence of low PCS %	X^2^(df=2)	P value
***T0***	–	–		–	–	–		–
***T1***	–	–		–	–	–		–
***T2***	–	–		–	–	–		–
***T3***	–	–		–	**Age**	–	6,242	0,001
	–	–			Young	12		
	–	–			Adult	21		
					Old	78		
***T4***	–	–		–	–	–		–
**LOWER GRADE GROUP**								
**Mental Component Score**					**Physical Component Score**			
	**Factors Associated**	**Prevalence of** **low MCS %**	**X^2^** **(df=1)**	**P value**	**Factors Associated**	**Prevalence of** **low PCS %**	**X^2^** **(df=1)**	**P value**
***T0***	–	–		–	–	–		–
***T1***	–	–		–	–	–		–
***T2***	–	–		–	**Adjuvant Treatments**		6,232	0,013
	–	–		–	Yes	74		
	–	–		–	No	46		
	–	–		–	**Language**		5,021	0,025
	–	–		–	Deficit	90		
	–	–		–	Normal	61		
***T3***	**Adjuvant Treatments**		10,022	0,002	**Adjuvant Treatments**		10,092	0,001
	Yes	80			Yes			
	No	37			No			
	**Language**		6,025	0,014	**Language**		3,869	0,049
	Deficit	79			Deficit	90		
	Normal	45			Normal	61		
***T4***	**Language**		10,254	0,007	**Language**		7,350	0,014
	Deficit	71			Deficit	79		
	Normal	14			Normal	52		

For each time points (t0: pre surgery; t1:1 month after surgery; t2:3 months after surgery; t3. 6 months after surgery; t4: 1 year after surgery) and for each group (High grade glioma in the upper part of the table; Low grade glioma in the lower part of the table) factors associated with low Mental (left part) and Physical (right part) component of the Health Related Quality of Life (HRQoL) was reported. Blank cells: no factors were statistically associated with the prevalence of HRQoL.

df= degree of freedom; *X^2^*= *chi square value.*

## Results

### Prevalence of Cognitive Deficits

In order to better describe the sample and to collect data about the cognitive and neurological status of the patient we calculated the prevalence of deficits in HGG and LGG patients (**Figure 1A**) and in LGG patients that underwent the adjuvant treatment *vs* LGG patients that were not treated (**Figure 1B**).

Compared to LGG patients, HGG patients had a higher prevalence of preoperative language (44% of HGG *vs* 20% of LGGs) and memory (44% of HGGs *vs* 23% of LGGs) impairment. At the first time-point after surgery (1 month) the prevalence of cognitive deficits was similar in both groups in all the five domains assessed. Three (t2) and six months (t3) after surgery the prevalence of some deficits was higher in the HGG group with respect to LGG group, specifically: at t2, language (HGG 46% *vs* LGG 28%) and attentive/executive deficits (HGG 27% *vs* LGG 13%); at t3, language (HGG 53% *vs* 28%), attentive/executive deficits (HGG 31% *vs* 14%), apraxia (HGG 7,5% *vs* LGG 0%) and motor deficit (HGG 15% *vs* LGG 4%). One year after surgery LGG and HGG differ only for language deficits (HGG 42% *vs* LGG 16%), motor deficits (HGG 14% *vs* LGG 5%) and memory deficits (HGG 0% *vs* LGG 14%). Overall, data seems to suggest that HGG patients had a higher prevalence of deficits compared to LGGs at specific time points, except for memory deficits, recorded at 1 year (**Figure 1A**).

Moreover, we compared patients treated with adjuvant therapies (LGGt) with those not treated (LGGnt) in the LGG group. There was a difference between the two groups from the t1 time point onward. Specifically, at t1 (1 month after surgery and before starting the adjuvant therapies) the LGGt group had a higher prevalence of attentive/executive (28% *vs* 9%) and motor deficits (27% *vs* 5%); than LGGnt group. At t2 and t3 (3 and 6 months after surgery) the LGGt group showed a higher prevalence of language (t2 36% *vs* 23%; t3 42% *vs* 18%), memory (t2 35% *vs* 21%; t3 28% *vs* 18%); apraxia (t2 and t3 8% *vs* 0%) and motor deficits (t2 17% *vs* 0%; t3 10% *vs* 0%) with respect to the LGGnt group. One year after surgery, prevalence of language (30% *vs* 7%) and motor deficits (11% *vs* 0%) was higher in the LGGt group than in LGGnt group. (see **Figure 1B**).

### Prevalence of Mood Disorders and of Low Health Related Quality of Life

For each time point, the prevalence of pathological score in HADS and in SF36 questionnaires for (Physical (PCS) or Mental (MCS) component of HRQoL was separately presented, for both HGGs and LGGs (**Figures 2** and **3A**) and for LGG patients that underwent adjuvant treatments *vs* LGG patients that were not treated with adjuvant therapies (**Figures 2** and **3B**).

#### Prevalence of Mood Disorders (MD)

MD were detected in both HGG and LGG patients at each time point that was assessed (**Figure 2A**). Before surgery (t0) and at 1(t1), 3(t2) and 12(t4) months after surgery no differences were found in the prevalence of MD between LGG and HGG patients. At these time points, prevalence of MD was as follows: (t0) 31% LGG *vs* 48% HGG; (t1) 54% LGG *vs* 53% HGG; (t2) 54% LGG *vs* 64% HGG; (t4) 45% LGG *vs* 63% HGG. Only 6 months after surgery (t3) we found there to be a prevalence of MD that was higher in HGG than in the LGG group (74% HGG *vs* 48% LGG) (**Figure 2A**). Following 3 months after surgery the prevalence of MD was higher in those LGG patients submitted to radiotherapy and chemotherapy (t2 72% *vs* 43%; t3 73% *vs* 33%; t4 68% *vs* 30%), with respect to the patients in which a “wait and see” approach was adopted (**Figure 2B**).

#### Prevalence of Low HRQoL

Before surgery about 60% of LGGs patients and 70% of HGGs patients showed a low score in both the mental (MCS) and physical (PCS) components of the HRQoL questionnaire (**Figure 3A**). At 1 month after surgery (t1), the prevalence of low HRQoL (for both mental and physical components) was around 80% in both groups (80%MCS and 84%PCS in LGG *vs* 78%MCS and 80%PCS in HGG; see **Figure 3A**); at 3 and 6 months after surgery, the prevalence of low scores in both components of HRQoL was around 65% (at t2) and 57% (at t3) in LGG patients (MCS t2: 63% t3 55%; PCS: t2 68%; t3 60%) and around 68% (at t2) and 70% (at t3) in HGG (MCS: t2 65%, t3 63; PCS: t2 72%; t3 77%), reaching the lowest percentage at 1 year after surgery in both groups (24%MSC and 30% PSC in LGGs *vs* 46% for both components in HGG) (**Figure 3A**). It is of note that there are no difference that were statistically significant, between the LGG and HGG groups.

Notably, in LGG patients, the prevalence of low HRQoL was significantly higher in those who underwent adjuvant treatments (radiotherapy/chemotherapy) in comparison to those who were just followed-up: at 3 and 6 months for the Physical and Mental components of HRQoL (PCS 81% *vs* 59%; MCS 73% *vs* 56% at t2; PCS 87% *vs* 45%: MCS 82% *vs* 40% at t3;) and also at 1 year after surgery for the Mental component only (33% *vs* 19%).

### Variables Associated With Mood Disorders and Health Related Quality of Life

#### LGG Patients

##### Mood Disorders

At admission, MD was associated with the occurrence of language deficits while at 1 month after surgery, MD was associated with motor deficits (MD documented in 60% of patients with language deficits and in 88% of patients with motor deficits, in comparison to 24% and 50% of patients who did not experience language or motor deficits). During the subsequent course of the disease (from 3 months (t2) to 6 months (t3) after surgery), the prevalence of MD was associated with language deficits (t2:80% of patients with deficit *vs* 44% of patients without; t3:72% *vs* 39%), and with the administration of adjuvant treatments (t2: 70% of patients who underwent to adjuvant treatments *vs* 40% of those who were not; t3 74% *vs* 32%; At 12 months after surgery (t4), we found significant associations again with language deficits (80% of patients with deficit *vs* 37% of patients without) and adjuvant treatments (69% who underwent to adjuvant treatments *vs* 30% of those who were not), but also with attentive/executive deficits (100% of patients with deficit *vs* 67% of patients without) (**Table 3**).

##### Health Related Quality of Life

At admission as well as at 1 month after surgery, low scores on the mental (MCS) and physical (PCS) components of HRQOL were not associated with any variable. On the contrary, at 3 months after surgery the low score in the Physical component of HRQoL was associated with language deficits (recorded in 90% of patients with deficits *vs* 61% of patients without) and undergoing adjuvant treatments (74% *vs* 46%); at 6 months after surgery low scores for both Mental and Physical components were associated with adjuvant treatments (PCS: 80% *vs* 36%; MCS: 80% *vs* 37%) and language deficits (PCS: 90% *vs* 61%; MCS: 79% *vs* 45%). Finally, at 12 months after surgery patients with language deficits showed a higher prevalence of low scores in both Physical and Mental components (PCS: 79% *vs* 52; MCS 71% *vs* 14%) (see **Table 4** for details).

#### HGGs Patients

##### Mood Disorders

At admission, 1 month and 12 months after surgery, MD in HGGs were not associated with any variable. At 3 months after surgery, MD were associated with language deficits (recorded in 84% of patients with deficits *vs* 54% of patients without) and with the affected hemisphere (70% in patients with a left hemisphere tumor *vs* 32% in those with a right hemisphere tumor). At 6 months after surgery along with language deficits (MD recorded in 90% of patients with language deficits *vs* 48% of patients without), MD was also associated with attentive/executive deficits (100% *vs* 67%) and older age (67% in patients >65 years *vs* 20% in 25-35 year old patients *vs* 33% in 36-65 year old patients) (**Table 3**).

##### Health Related Quality of Life

Both components of HRQoL (MCS and PCS) were associated with older age (78% in patients >65 years; 12% in 25-35 year old patients; 21% in 36-65 year old patients) (**Table 4**).

## Discussion

This work addresses the prevalence and time course of MD and HRQoL in LGGs and HGGs patients. It provides new insight into the secondary effect of the brain tumor on MD and HRQoL, through assessment of the clinical or functional factors that possibly contribute to their occurrence during the first year of the disease (from the pre surgical period up to 12 months after diagnosis).

The analysis was performed on a group of patients treated for a glioma who were stable for the disease during the observation time of the study, without previous or current psychiatric disorders and in a clinical condition allowing them to successfully complete the questionnaires provided. This was done to rule out the possible influence exerted by the progression of the disease and previous psychological disorders on the prevalence of MD and HQoL disorders recorded during the observation period.

The first interesting finding is the surprisingly high prevalence of poor HRQoL and MD symptoms since diagnosis, in both HGG and LGG group. Moreover, the prevalence of low HRQoL did not differ between HGG and LGGs, confirming previous observation (16) reporting that quality of life is not affected by the tumor grade per sè but by the stability of the disease over time. This hypothesis may be supported by the fact that in both groups (HGG and LGG) the prevalence of poor HRQoL recorded at 1 year after surgery was lower than at 6 months, possibly because the absence of progression allows the patient to recover from the adopted treatments. On the contrary, by the 6 month following surgery, LGG patients showed a significantly lower prevalence of MD than the high-grade patients which may suggest a potential effect of the tumor or of the different treatment approaches adopted for the management of the disease or a combined effect of the two variables. Despite the effect of treatment, it is difficult to disentangle this within the HGG group given that all patients are generally submitted to adjuvant treatments, and analyses performed within the LGG group seems to support the effect exerted by the adjuvant treatment on the prevalence of MD and HRQoL. In fact, significant differences were found in the prevalence of MD and poor HRQoL between LGGs patients who underwent adjuvant treatments at the 3 month post surgery time point and the patients that were submitted to observation only.

Bivariate analysis confirms the hypothesis that tumor aggressiveness and the adoption of therapy affect patient psychological status and the quality of life, and also highlights the effect exerted by the cognitive deficit. HGGs patients developing language deficits or affected by left hemisphere tumors experienced a higher prevalence of MD at 3 months after surgery, while at 6 months the cognitive deficits (language, attentive/executive) and older age (>65-75 years) were associated with MD. In the LGG group, the patient’s cognitive status, specifically motor, language and attentive/executive deficits, was the main factor associated with the prevalence of MD at each time point, and that influenced the course of both components of HRQoL, starting of 3 months after surgery. From the same time point, adjuvant treatments were also associated with MD and poor HRQoL.

Our findings differ from previous studies reporting no difference in mood symptoms among patients with different WHO tumor grades and no association between cognitive performance and MD (36). However, in these studies, the assessment of MD was conducted either before surgery or immediately after (7, 21, 23, 37) or at a much later stage of the follow-up, in the outpatient clinic (12, 22) in addition, by using less sensitive neuropsychological tests. As reported by Rooney and colleagues (24) it is in the intervening period, i.e. 3-6 months after surgery, the tumor grade might be expected to mediate differences in mood; it is in this period that patients can react differently to their different prognoses and to different treatment approach but also to functional recovery.

Our findings are in line with previous evidence reporting that when a detailed neuropsychological testing is used, MD is associated with cognitive impairment (38, 39). This is not surprising considering the impact that cognitive dysfunction exerts on patient’s functional performance, leading to an impairment of self-sufficiency. Regarding the lack of associations between specific single functional factors and poor HRQoL in HGG patients, we theorizes that these results may be related to the complex interplay of multiple deficits typically observed in these patients, which prevent the identification of the role of each individual factor. Moreover, HGG patients are generally older and submitted in all cases to adjuvant treatments. In itself, this may lead to a more difficult recovery and prevent patients from reaching a quality of life that is comparable to that experienced in the pre-operative phase.

In contrast to previous findings that report a relevant role of the right hemisphere in predicting the occurrence of MD (7, 12, 24) and low HRQol (40, 41), our data suggested that at 3 months after surgery, the prevalence of MD was associated with the presence of a tumor in the left hemisphere. This may be explained by the fact that the occurrence of MD was associated with that of language impairment (usually observed in patients with a tumor in the dominant hemisphere), which is associated with functional limitations and impaired the self-sufficiency.

These results point out the need to preserve the patient’s functional integrity using the best available treatments (surgical techniques and adjuvants therapies) (42–45).

The absence of significant association with clinical variables (such as histology, IDH-status, or tumor location) does not exclude that tumor-related factors play an indirect role in the etiology of MD or poor HRQoL. However, given that the prevalence of MD and HRQoL changes over time (showing a decrease especially at the long-term compared to baseline), whereas the tumor location, IDH-status, extent of resection and adopted treatments did not change, suggests that prevalence of MD and HRQoL overtime is due to additional reasons. These premises, supports a multifactorial genesis of the mood status and the subjective perception of how the disease affects an individual patients’ quality of life; it seems clear that a wide variety of factors are relevant over the course of the disease and at the individual patient level, all possibly contributing to mood status and the QoL experience.

Additional longitudinal studies are needed to better understand the patient’s psychological status and quality of life, especially for patients affected by severe cognitive deficits (for example language impairment) that make it hard to administer standardized questionnaire on mood or quality of life. The first unavoidable limitation is related to the difficulty of collecting a complete longitudinal setting of data after one year due to a high prevalence of disease progression and severe cognitive morbidity in HGG. Moreover, although the relationships between MD, HRQoL and adjuvant treatments seems to be evident, it is hard to definitively disentangle the effects of chemo/radio-therapy treatments from the role of the tumor itself, given that all the HGG patients undergo radiotherapy with concomitant adjuvant temozolomide. Further research that accounts for confounding factors is necessary in order to define specific treatment-related symptoms and their contribution to decreased HRQOL.

Other issues, such as individual differences (personality), the social context and the relationship between the HRQoL and the emotional reactions of caregivers and patients, the sense of uncertainty about treatment and life expectancy need to be also explored. The implementation of qualitative research (such as focus group studies) into research practice may allow us to obtain this crucial information also about patients affected by severe deficits or with relapse, in order to understand other psychological aspects contributing to the poor prognosis and poor HRQoL. More specifically, patients vulnerable to MD or decline in HRQoL might be identified at an earlier stage, which would allow for personalized and timely psychological interventions.

Despite these limitations, some conclusions can be drawn for HRQoL and MD in brain tumor patients. Specifically, our data show that the prevalence of MDs and low HRQoL is unexpectedly high, especially during the first months after surgery and in patients undergoing adjuvant treatment. As a matter of fact, the assessment of MD and HRQoL as primary or secondary end points must be systematically addressed in glioma patients because of their intrinsic significant prognostic value. An improvement in the patient’s well-being with in turn increase the overall survival.

## Data Availability Statement

The data analyzed in this study is subject to the following licenses/restrictions: Data will be promptly available upon reasonable request to the authors. Requests to access these datasets should be directed to antonellaleonetti80@gmail.com.

## Ethics Statement

The studies involving human participants were reviewed and approved by Humanitas Research Hospital Irb1299. The patients/participants provided their written informed consent to participate in this study.

## Author Contributions

Conceived the study: AL, GP, and LB. Selected patients, directed, and executed the surgical procedure and the intraoperative brain mapping: BL. Acquired data, analyzed data: AL, GP, MRo, FL, LB, HH, LV, MC, LG, MR, and ST. Produced figures: AL, LV, MRi, and HH. Discussed and interpreted the results: all the authors. Wrote the first draft of the paper: AL, GP, LB, and GC. All authors contributed to the article and approved the submitted version.

## Funding

This work was supported by grant from “Associazione Italiana per la Ricerca sul Cancro (AIRC)” 18842 to LB.

## Conflict of Interest

The authors declare that the research was conducted in the absence of any commercial or financial relationships that could be construed as a potential conflict of interest.
